# Anti-infliximab antibodies are already detectable in most patients with rheumatoid arthritis halfway through an infusioncycle: an open-label pharmacokinetic cohort study

**DOI:** 10.1186/1471-2474-12-12

**Published:** 2011-01-13

**Authors:** Bart JF van den Bemt, Alfons A den Broeder, GJ Wolbink, Yechiel A Hekster, Piet LCM van Riel, Bart Benraad, Frank HJ van den Hoogen

**Affiliations:** 1 Department of Pharmacy, Sint Maartenskliniek, Nijmegen, The Netherlands; 2 Department of Rheumatology, Sint Maartenskliniek, Nijmegen, The Netherlands; 3 Department of immunopathology, Sanquin Research, Amsterdam; 4 Department of Pharmacy, Radboud University Nijmegen Medical Center Nijmegen, Nijmegen, The Netherlands; 5 Department of Rheumatology, Radboud University Nijmegen Medical Center, Nijmegen, The Netherlands

## Abstract

**Background:**

This study in patients with rheumatoid arthritis (RA) treated with infliximab describes prospectively the course of (anti)infliximab levels within an infusioncycle to assess at what moment patients develop low/no infliximab trough levels and/or detectable anti-infliximab levels.

**Methods:**

Infliximab treated RA patients were included in this descriptive open-label cohort study. During one infusioncycle (anti-)infliximab levels were assessed just before and one hour after infusion, and subsequently at 50%, 75% and at the end of the infusioncycle (pre-infusion).

**Results:**

27 patients were included. The median infliximab levels decreased from 77.0 mg/l (p25-p75: 65-89) one hour after the infusion to pre-infusion levels of 0.0 mg/l (p25-p75: 0.0-3.1). In 7 (26%) patients pre-infusion anti-infliximab antibodies were detected; these antibodies were already present halfway through the infusioncycle in 5 of the 7 individuals. Patients with detectable pre-infusion anti-infliximab antibodies have significantly more often low/no infliximab levels (< 1 mg/l) halfway trough the infusioncycle (in 5/7 patients) compared to patients without detectable pre-infusion anti-infliximab antibodies (0/20 patients, p < 0.001).

**Conclusions:**

Most anti-infliximab forming patients have detectable anti-infliximab antibodies halfway through an infusioncycle, which implies that these patients are exposed to nontherapeutical infliximab levels during more than halve of their infusion cycle. As none of the patients without anti-infliximab antibodies had no/low-infliximab levels halfway through the infusioncycle, the presence of pre-infusion anti-infliximab antibodies seems a sensitive and specific predictor for no/low infliximab-levels

## Background

Rheumatoid arthritis (RA) is a chronic autoimmune disease characterised by inflammation of synovial tissue leading to progressive articular cartilage and bone destruction. To prevent progression of joint damage and functional disability, early introduction of effective disease modifying antirheumatic drugs (DMARDs) is considered to be essential in the treatment of patients with rheumatoid arthritis (RA). Besides traditional DMARDS like methotrexate, tumour necrosis factor (TNF) antagonists have been proven to reduce disease activity, suppress radiographic joint damage and decrease functional disability in patients with recent onset [1,2] and established rheumatoid arthritis (RA)[3,4]. About 40-60% and 20-40% of the patients met the American College of Rheumatology (ACR) 50% and 70% improvement criteria respectively [5], compared to placebo improvement percentages of 7-11% (ACR50) and 2-4% (ACR70).

However, these results also implicate that up to 60% of patients with RA do not reach the clinical relevant 50% improvement. Therefore, non-responders (both primary as secondary non-responders) should be identified as early as possible. Firstly, a shorter period of high disease activity minimises chances of joint destruction [6]. Also treatment with TNF antagonist is associated with considerable costs. Finally there is ongoing debate on their safety and possible dose related adverse effects [7,8].

Because valid prediction models are not available at this point, close monitoring of individual disease activity and adapting the treatment (dose) is the first available step to improve the efficacy of RA-therapy [9,10]. Although disease activity guided treatment is a valuable instrument, this strategy cannot distinguish between patients who improve through the pharmacological effect of infliximab or patients who's improvement in disease activity is caused by co-medication, expectation bias or more importantly the natural course of the disease (regression to the mean) [11].

Pharmacokinetic data with infliximab indeed show that some patients achieve improvement and low disease activity during therapy with infliximab, although this response could most likely not be attributed to infliximab as these patients had no- or low-infliximab trough levels. These reduced levels could partially be explained by the formation of human antichimeric antibodies (HACAs) which occurs in 8% to 43% of the RA patients [12-14]. The formation of antibodies against infliximab has been associated with altered infliximab pharmacokinetics and reduced serum infliximab concentrations over time in patients with RA [12,13].

Clinically, it is relevant to know whether patients with serum trough anti-infliximab antibodies also have these antibodies present early in a treatment cycle or whether they appear only at the end of a treatment cycle. Patients with "early" anti-infliximab detectable antibody formation would have a long window wit nontherapeutical levels of infliximab. The alternative scenario, appearance of HACA's predominately at the end of the infusion cycle would be less important as adequate infliximab levels would be present during the majority of time between infusions. However, until now, it is unknown what the relationship is between trough anti-infliximab antibody levels and (anti-)infliximab antibody throughout the treatment cycle.

This study therefore prospectively describes the course of (anti)infliximab levels within an infusioncycle in patients with rheumatoid arthritis in order to assess at what moment patients develop low/no infliximab trough levels and/or detectable anti-infliximab levels.

## Methods

### Patients

Patients with RA, according to the ACR 1987 revised criteria, treated at the Sint Maartenskliniek (Nijmegen, The Netherlands) for at least 3 months with 3 mg/kg infliximab (irrespective of dose frequency) were included in this observational, descriptive open-label pharmacokinetic cohort study. No other inclusion or exclusion criteria were used. In the Sint Maartenskliniek all RA patients receive 3 mg/kg infliximab, with dose intervals adjusted to patient's disease activity. Patients were treated according to the local disease activity guided protocol, When a patient does not reach low disease activity on 3 mg/kg/4 wks the patient is switched to another DMARD or biological.

### Study protocol

Patients were enrolled between February and April 2008. Ethical approval was obtained from the Ethics Committee Nijmegen-Arnhem and all participants gave written informed consent before screening. A standardized chart review form was used to collect data on demographics, previous medication and clinical benefit of infliximab.

### Pharmacokinetic and pharmacodynamic assessment

Serum samples for the measurement of (anti-)infliximab levels were collected during one treatment cycle at five time-points: one hour prior to the first infusion, one hour after the infusion, at 50% and 75% of the infusioncycle, and just before the next infusion. The Disease Activity Score (DAS28) was assessed at the same time points excluding the time point just after the first infusion (as this DAS-score is equal to the DAS-score prior to the first infusion).

Infliximab- and anti-infliximab antibody levels in serum were determined by an enzyme-linked immunosorbent assay and a radioimmunoassay respectively [13,15]. We categorized serum trough levels in low (< 1 mg/l), medium (1-5 mg/l) and high (> 5 mg/l) levels. In contrast to the lower limit (1 mg/l), less information is available about the maximum desirable infliximab serum trough level. Therefore, we arbitrary choose that serum trough levels above 5 mg/l are high levels, which is 5 times the minimum serum trough level and 3.3 times the average serum trough level. Previously, Wolbink et al [15] used tertiles to categorize serum levels at 14 weeks in low, medium and high levels, also categorizing serum trough levels above 5 mg/l as high.

### Statistical analysis

Descriptive statistics were provided using mean (+/- SD) or median (p25-p75) values depending on the (non-) parametric distribution of the variables. We used Mantel-Haenszel x^2^-tests to evaluate differences in proportions, and Student's t-tests to evaluate differences in means. Non-parametric variables were analyzed using the two-sample paired sign test. The threshold for significance was set at p = 0.05. To test whether the relationship between the formation of anti-infliximab antibodies and inflximab serum trough levels is influenced by confounders, the baseline variables of anti-infliximab formers and non-formers were compared in a univariate analysis and in a multivariate logistic association model using the presence of no/low-infliximablevels as dependent variable, antibody formation as central determinant and all variables with a significant univariate association with both the dependent and independent variable of p < 0.1 as potential confounder. Confounders were kept in the model when the beta for the central determinant changed with > 10% after addition of the confounder

## Results

Twenty-seven patients were enrolled in the study; their demographic and clinical data at baseline are summarized in table 1.

**Table 1:** Baseline characteristics of patients

	n = 27
Age (yrs)	61.6 ± 10.0
Woman (n,%)	15 (56)
Median disease duration (yrs, p25-p75)	11.2 (4.2- 17.4)
Median number of previous DMARDs (n)	2.5 (2.0- 3.3)
Previously treated with another biological (n, %)	3 (11%)
Rheumatoid factor positive (n,%)	21 (78)
Duration of infliximab therapy (yrs)	3.7 ± 2.3
Interval infliximab infusions (wks)	6.8 ± 2.0
Disease Activity at baseline	
Remission (n,%)	7 (26)
Low disease activity (n,%)	6 (22)
Moderate disease activity (n,%)	11 (41)
High disease activity (n,%)	3 (11)
DMARD at baseline (n,%)	23 (85)
Methotrexate (n,%)	17 (63)
Dose (mg/week)	16.8 ± 5.5
Azathioprine (n,%)	4 (15%)
Prednisone at baseline (n, %)	5 (19%)
Dose (mg/day)	5.8 ± 1.8

Variables are expressed as mean ± SD unless stated otherwise.

wks = weeks; mths = months; yrs = years

### (Anti) Infliximab concentrations

**Figure 1:**
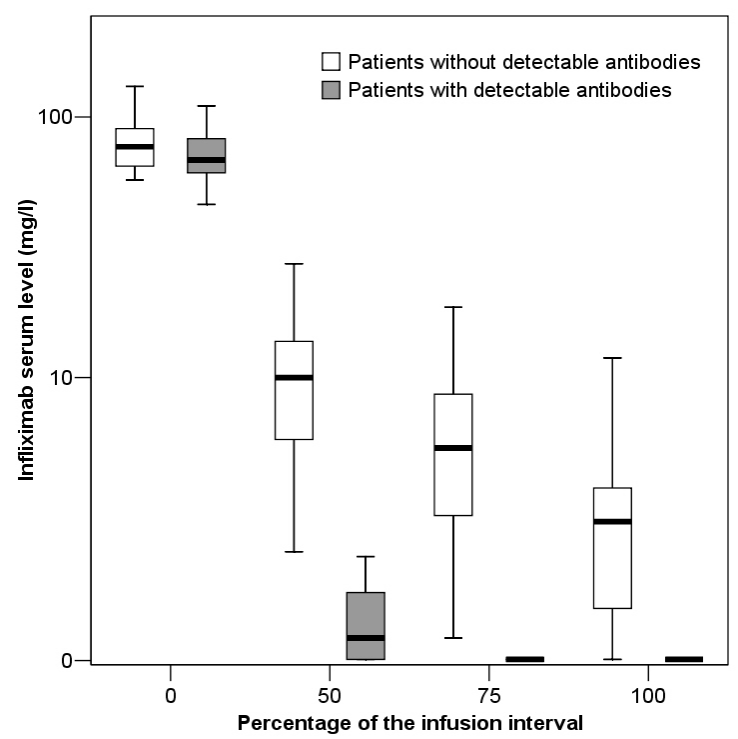
**Serum trough infliximab levels during the infusioncycle in antibody-forming and non-anti-body forming RA patients**.

**Figure 2:**
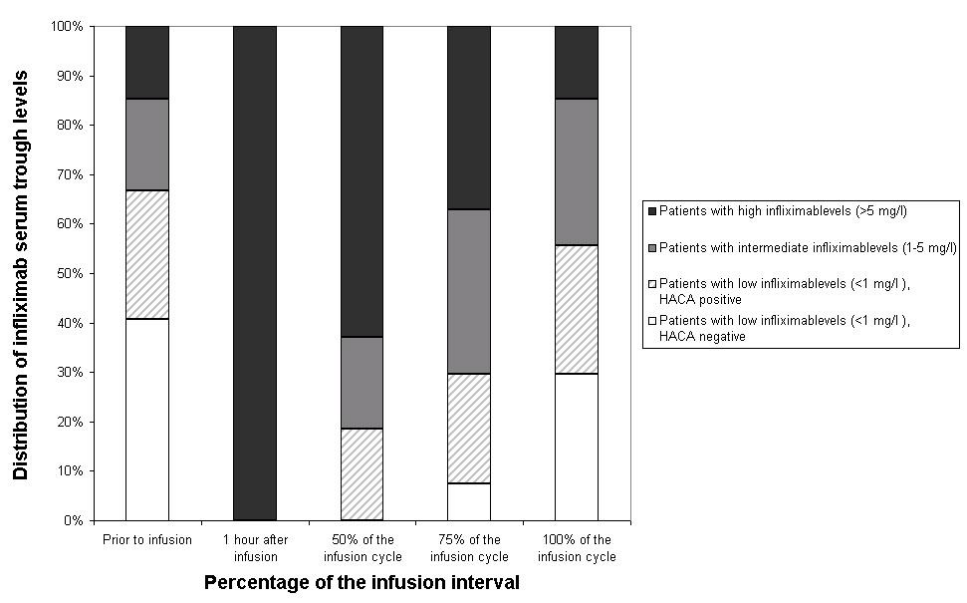
**Distribution of infliximabserum trough levels and HACA-formation during the infusion interval**.

Table 2 shows the median infliximab levels and the decrease of infliximab serum trough levels during the infusioncycle. In 7 (26%) patients anti-infliximab antibodies were detected just prior to the next infusion. These antibodies were already present at 50% of the infusioncycle in 5 of the 7 individuals. Infliximab serum levels during a single infusioncycle categorized as patients with and without detectable anti-infliximab antibodies are depicted in figure 1 and 2. At 50% of the infusioncycle 5/7 (71% ± 33%) of the patients with HACAs just before the next infusion had low infliximab serum levels (< 1 mg/l) which was significantly more frequent compared to 0/20 of the non-antibody forming patients (p < 0.001).

**Table 2:** Median and distribution of infliximab serum trough levels

	Prior to infusion	1 hour after infusion	50% of the infusioncycle	75% of the infusioncycle	100% of the infusioncycle
**Median infliximab levels**					
Median infliximab levels (p25-p75)	0.6 (0.0 - 3.1)	77.0 (65-89)	5.9 (1.5-13)	2.7 (0.2-5.7)	0.0 (0.0-3.1).

Anti-infliximab antibodies were significantly (p < 0.01) more frequent in rheumatoid factor negative patients (4 of 6 patients (67%)) compared to rheumatoid factor positive patients (3 of 21 patients (14%)). Patients with detectable antibodies were also significantly younger (mean age: 53.5 (± 13.6) years) compared to patients without detectable antibodies (mean age: 64.5 (± 6.7) years; p < 0.01). Other baseline variables were not associated with the formation of anti-infliximab antibodies.

Concomitant DMARDs were used in 6 (86%) of the patients with detectable anti-infliximab antibodies and in 17 (85%) of patients without anti-infliximab antibodies and remission or low disease activity was present in 4(57%) of the patients with detectable anti-infliximab antibodies compared to 9 (45%) patients without detectable antibodies. A logistic regression association model showed that age and the presence of rheumatoid factor did not act as confounder on the relation between anti-infliximab antibodies formation and infliximab serum trough levels.

### Course of the disease activity between two infusions

Disease activity (DAS28) and EULAR-disease activity classification percentages are shown in table 3. Disease activity was significantly (p = 0.03) higher prior to the next infusion than the disease activity halfway through the infusion. At the end of the infusion interval 11 (41%) patients showed low disease activity or remission, which tended to be lower compared to the 16 (59%) patients with low disease activity or remission halfway through the infusion interval (p = 0.06).

**Table 3:** Disease activity (measured by the DAS28) and EULAR-disease activity classification percentages during one infusion interval

	Prior to infusion	50% of the infusioninterval	75% of the infusioninterval	100% of the infusioninterval
Mean DAS28 (± SD)	3.3 (± 1.1)	3.0 (± 1.0)	3.4 (± 1.1)	3.4 (± 1.1)
Patients in remission (n, %)	7 (26%)	10 (37%)	6 (22%)	8 (30%)
Patients with low disease activity (n, %)	6 (22%)	6 (22%)	6 (22%)	3 (11%)
Patients with moderate disease activity (n, %)	11 (41%)	11 (41%)	14 (52%)	15 (56%)
Patients with high disease activity (n, %)	3 (11%)	0 (0%)	1 (4%)	1 (4%)

## Discussion

Our results indicate that anti-infliximab antibodies are frequently found in patients with low and moderate disease activity and that these antibodies are already detectable in most of these patients halfway through an infusioncycle. This implies that the presence of anti-infliximab antibodies at the end of an infusioncycle seems a good predictor for low infliximab-levels throughout most of the infusioncycle.

The internal validity of our study appears to be adequate. Although the number of patients in the present study is limited, this does not necessarily hamper the validity of this study as a higher number of patients would only increase precision. Confounding was considered because the presence of rheumatoid factor was related to both anti-infliximab formation and no/low infliximab levels, however a multivariate regression model could not demonstrate confounding.

The design of this study is not suitable to draw conclusions about the correlation between pharmacokinetic- ((anti)-infliximab levels) and the pharmacodynamic- (disease activity) parameters. Patients were treated according to the local disease activity guided protocol, which automatically excluded the majority of non-responders in this observational cohort. This could lead to a selected study population, in which pharmacokinetic parameters could not be correlated with non-response. Due to this selection bias, this study also can not be used to assess possible risk factors for the formation of anti-infliximab antibodies.

This study has two significant clinical implications. First, we found that in patients with low infliximab trough levels, the presence of serum trough anti-infliximab anti-bodies could be a specific and sensitive indicator for absence of serum infliximab level during at least half of the infusioncycle.

Secondly, our finding that one fifth of the patients treated with infliximab have already non/low infliximab-levels halfway through the infusion suggests that these patients do not benefit from infliximab at all. One could argue that the effect of infliximab therapy may be (partially) determined by peak levels or time integrated AUC rather than by minimal inhibitory concentration (MIC), implying that measuring serum trough levels is not indicative for clinical effect. This is however not likely as subcutaneous anti-TNF agents demonstrate similar efficacy without high peak serum levels [16]. However, this issue can only be clarified in an intervention study in which the dose of infliximab is tapered down and stopped in patients with anti-infliximab levels and/or suspected nontherapeutical infliximab levels.

In this study, we found an increase in disease activity at the end of the infusion interval. This could lead to an underestimation of the effect of infliximab when disease activity is only measured just before infusion. However, previous research demonstrated that fluctuations in disease activity also importantly and independently contribute to radiologic progression [17]. This implicates that anti-rheumatic drugs should keep disease activity at a stable, low level, and consequently keep structural damage to a minimum. These finding also implicates that observational studies comparing subcutaneous- (etanercept adalimumab) and intravenous- (infliximab, abatacept and tocilizumab) antirheumatic agents should be interpreted with caution when disease activity for intravenous agents is conveniently assessed at the end of an infusion interval while disease activity in other drugs is often assessed on different moments during a dosing interval.

## Conclusion

This study demonstrates that a substantial proportion of RA-patients treated with infliximab are already exposed to no/low-infliximab levels during more than halve of their infusion cycle. The presence of pre-infusion anti-infliximab antibodies could be used as a sensitive and specific predictor for no/low infliximab-levels halfway the infusioncycle.

## Competing interests

The authors declare that they have no competing interests.

## Authors' contributions

BvdB, AdB, GW, YH, PR, BB and FH conceived and designed the study. All authors were involved in carrying out the study and interpreting the results. BvdB performed the statistical analysis and drafted the manuscript. All authors read, commented on and approved the final manuscript.

## Pre-publication history

The pre-publication history for this paper can be accessed here:

http://www.biomedcentral.com/1471-2474/12/12/prepub
